# Congenital goiter in North of Iran: A case report

**DOI:** 10.1002/ccr3.3155

**Published:** 2020-08-20

**Authors:** Shahrokh Mehrpisheh, Daniel Zamanfar, Zahra Ghaffari, Azadeh Memarian, Farahnaz Nikkhah

**Affiliations:** ^1^ Neonatology Department Mazandaran University of Medical Sciences Sari Iran; ^2^ Pediatric Endocrinologist Diabetes Research Center of Mazandaran Mazandaran University of Medical Sciences Sari Iran; ^3^ Forensic Medicine Department Rasoul Akram Hospital Iran University of Medical Sciences Tehran Iran; ^4^ Rasoul Akram Hospital Clinical Research Development Center (RCRDC) Iran University of Medical Sciences Tehran Iran

**Keywords:** congenital goiter, congenital hypothyroidism, iodine, pregnancy diabetes

## Abstract

Congenital goiter (CG) is one of the rarest disorders observed in a newborn at birth diagnosed with hypothyroidism. Considering the simultaneity of pregnancy and baby's hypothyroidism at birth, the goiter can be caused by diabetes during pregnancy and hypothyroidism emergence in the baby.

## Graphical abstract

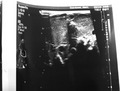

## 1 INTRODUCTION

Goiter is one of the rare causes of head and neck masses in newborns, which is observed in one case per 40 000 births.^1^ This complication in infants is often associated with congenital hypothyroidism (CH), which can be caused by a hereditary problem or some nonhereditary causes such as monogenesis, or maternal antibodies and antithyroid drugs passing through the placenta.^2^ In a few cases, the disorder can be caused by excessive iodine that mother intakes.

Congenital hypothyroidism can be diagnosed during pregnancy or at birth.^3^ Sometimes the thyroid size at birth is not too large to be noticed, and sometimes its largeness can cause severe respiratory symptoms at birth, requiring intubation.^4^ Hard vaginal delivery is observed in cases with a large thyroid. The most important disorder associated with CG is hypothyroidism and if it is left untreated leads to many disorders and consequently, cretinism.^5^ For this reason, the rapid diagnosis of CG, and the examination of thyroid hormone status, is highly important for preventing the complications resulting from thyroid enlargement as well as hormonal changes.

Ultrasonography is the main diagnostic method of CG, which often displays homogeneous size in the thyroid. Furthermore, ultrasonography can examine thyroid pressure on other neck organs as well as their displacement.^5^ In some cases, thyroid enlargement is detectable in ultrasound examinations during pregnancy.^6^ In some centers, after the diagnosis of goiter in the womb, fetal thyroid hormones are examined and the fetus is treated, if necessary.^7^ Care should be taken in case of a goiter diagnosis during pregnancy, and the medical team should be ready to the care for the baby at birth. If there is no severe respiratory complication, levothyroxine is prescribed. In this regard, the thyroid enlargement gradually disappears. Moreover, neonates with CG and CH should be monitored regularly and their dosage should be adjusted for thyroid hormone status as well as thyroid‐stimulating hormone (TSH).^8^ Because of the rareness of this disease, few cases have been reported in the world. The present study introduced a case of cervical mass at the birth in a neonate in the north of Iran followed up by a CG diagnosis.

## 2 CASE REPORT

Our patient was a 38‐week‐and‐5‐day‐old boy born through cesarean due to fetal distress. He was weighted 2580 g at birth, first‐minute and fifth‐minute Apgar were 9 and 10, and amniotic fluid was transparent. The infant was admitted to the NICU ward for tachypnea and respiratory distress. The examination of the infant showed swelling in the anterior neck, which was mildly palpable and no subcutaneous emphysema was seen. An intercostal retraction was seen on both sides of the chest and there was no deformity. The lungs were clean. No murmurs or excessive sound was heard on cardiac examination. Other newborn examinations were also normal.

**Figure 1:**
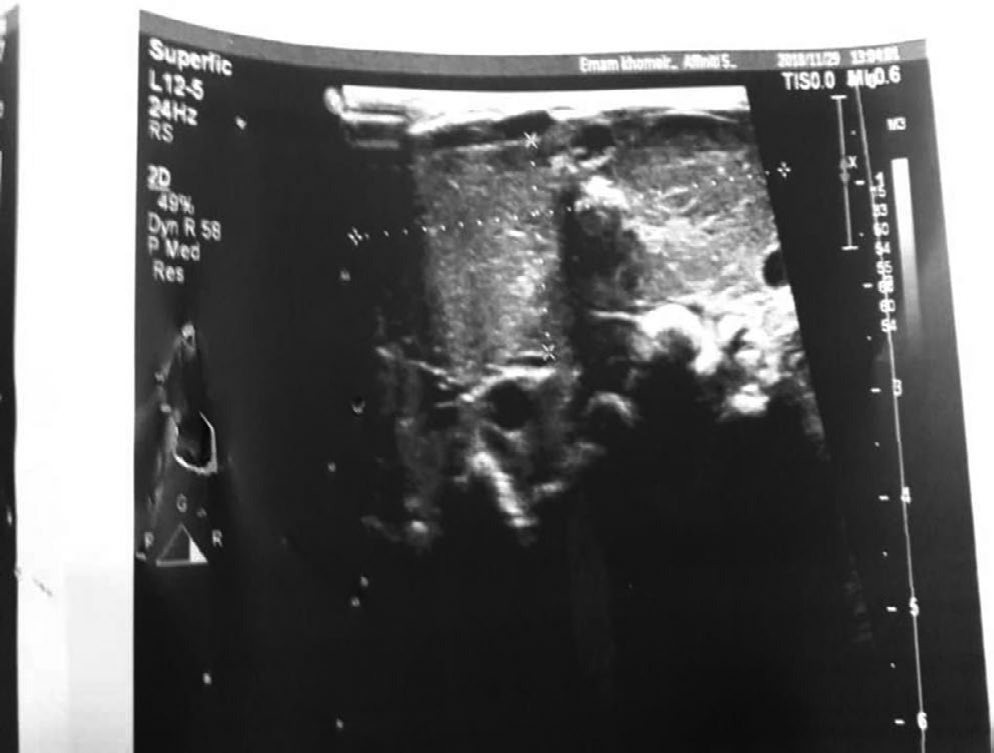
Ultrasound neck mass image

The infant underwent ultrasonography for the diagnosis of a neck mass. Ultrasound showed a homogeneous echogenic soft tissue mass with markedly increased vascularity that was symmetrically on the chip. The mass was 55 × 20 mm. Goiter was diagnosed based on a mass profile (Figure 1). Thyroid hormones were measured to evaluate thyroid function (results of neonatal screening for CH) T4 = 4.39 micg/dL, FT4 = 1.1 ng/dL, FT3 = 4.1 pg/mL, and TSH = 15.9 m IU/L, respectively (Table 1). Parents did not agree for any further genetic investigations.

**Table 1:** Hormone analysis

Test	Result	Unit	Normal range
T4	4.39	micg/dL	4.2‐14
TSH	15.9	m IU/L	0.5‐5.5
FT3	4.1	Pg/mL	2.2‐4.2
FT4	1.1	ng/dL	0.8‐1.7

The infant's mother was 27 years old from Sari, Mazandaran, Iran. She had no past medical history of thyroid or any autoimmune disease. The baby was born from her third pregnancy. The previous two pregnancies had resulted in miscarriage, and the baby was the mother's first living child. The cause of the mother's two previous miscarriages was not mentioned, and the presence of “goiter” in the two previous pregnancies was unclear. During pregnancy, the mother had gestational diabetes and underwent diet therapy. History did not detect any other problems during pregnancy. Postnatal maternal thyroid function was T4 = 8.4 micg/dL and TSH = 1.63 m IU/L. The mother had consumed iodine‐containing salt during pregnancy. Received supplements and vitamins during pregnancy were identified and did not contain iodine. Ultrasounds were normal during pregnancy and showed no mass at the site of the fetal neck. The baby's parents were not relative.

Immediately after the diagnosis, levothyroxine was administered orally at the time of diagnosis for 40 micrograms per day. The infant was followed up monthly and TSH and free T4 levels were measured at each visit, and the dose of the drug was adjusted based on hormone levels. The baby's TSH was normal in the second to fifth months but increased again in the sixth month when the drug was modified. After 6 months of the follow‐up, ultrasound was performed for the infant and the size of the normal thyroid was reported. After 6 months, the baby was 6500 g in weight. The infant's growth status was normal during the 6 months and equaled the 25th percentile.

## 3 DISCUSSION

Congenital goiter is a rare disease at birth and during pregnancy. This complication can cause polyhydramnios, tracheal and esophageal deformities during pregnancy, decreased intrauterine growth, and even fetal death.^9^ This disease mostly arises from the primary hypothyroidism in the fetus when the mother does not take antithyroid drugs, excessive iodine intake, or have no Graves’ or Hashimoto's disease. In general, CG is observed in about 10% of CH cases.^10^, ^11^ At birth, a fetus with goiter may have severe respiratory symptoms, which requires intubation.^4^

According to the study of Mirsaeid Ghazi et al, the CG reported in Iran was seen in only one case,^12^ which was related to a 34‐week fetus with goiter. The mother was 33 years old and had no history of thyroid disease. The reported fetus was born from the third pregnancy. Neonates in both previous pregnancies had CG. The first baby, born after a cesarean section due to the poor fetal status, had respiratory problems and died immediately after the birth due to thyroid pressure on the trachea. The second baby had CG and CH, and was accurately treated. She was 8 years old at the time of reporting and had a normal physical and educational status, but despite appropriate treatment, she still had a goiter. At the time of the third maternal pregnancy and considering previous pregnancies at 34 weeks of pregnancy, fetal thyroiditis was assessed by ultrasound, and goiter was observed. Maternal thyroid size and function were normal during pregnancy, and the fetus had no problems during the second ultrasound. However, amniocentesis was performed as requested by the parents and a dose of levothyroxine injected into the amniotic cavity. Levothyroxine injections were also given weekly in three successive weeks and the infant was finally born at 38 weeks. There was no clear thyroid enlargement at the birth, but radiography of knee did not show epiphyseal growth of femoral and tibial bones. The infant had been treated with levothyroxine and was examined on a monthly basis.^12^

Similar to the study of Mirsaeid Ghazi et al, the infant in the present study was born from the third pregnancy. The previous two pregnancies resulted in a miscarriage. Unfortunately, the age and the cause of miscarriage, and premiscarriage studies were unavailable, and we are not sure if previous embryos also had a goiter. Ultrasound scans revealed no thyroid enlargement during pregnancy and no lesions such as polyhydramnios. The mother did not have any prior thyroid disease before and during pregnancy and did not take any antithyroid medication but had gestational diabetes and underwent dietary treatment. Several studies have shown a relationship between gestational diabetes mellitus (GDM) and CH.^13^ A statistically significant association of CH was observed with birth defects, female gender, gestational age >40 weeks, and GDM. ^14^ In another population‐based case‐control study in Italy, the results also showed that GDM was a risk factor for CH.^15^ Few studies have investigated the presence of ThyAb in women with gestational diabetes mellitus (GDM), although CH appears to be a multifactorial problem depending on genetic and environmental factors in the fetus, it is not clear that in our infant, only GDM causes CG and CH.^16^ The finding of raised TSH in GDM fetus as compared to control shows that it could not be due to Immaturity of fetal HPT axis or transient hypothyroidism of prematurity which is not possible as gestational age was appropriate (37 weeks). There are studies which demonstrated that pregnant women with increased risk of GDM have a high prevalence of raised ThyAb [thyroid antibody] than observed in general women population. However, few studies have reported no association between diabetes in pregnancy and thyroid function [ThyAb) in pregnant women with GDM.^17^, ^18^

Another factor that may affect the incidence of CG in the studied neonate is the consumption of iodine‐containing salts and iodine intake beyond the standard limit. In Iran, due to the high prevalence of hypothyroidism, especially in the north part of the country, iodine‐containing salts were recommended to the people many years ago and most iodized salts were added. Although there have been few cases of maternal iodine intake in CG cases around the world, this should be taken into account. In one study, eight infants with CG whose mothers had high‐iodine intake were identified. Increasing iodine intake leads to an increase in intrathyroidal iodine concentration and a decrease in organogenesis and thyroid hormone. This mechanism, which occurs due to the preservation of life and the prevention of overproduction of thyroid hormone, can gradually decrease thyroid hormone and goiter.^19^ In another study, three newborns with CG were identified who were not initially positive for their cause, but further studies revealed that both mothers had taken high‐iodine herbal remedies.^20^

During pregnancy, the fetus uses maternal thyroid hormones at the beginning, and before the full formation of the thyroid gland and in ultrasound taken at 20‐36 weeks of gestation, thyroid size and cervical mass are examined. Measurement of thyroid hormones and TSH by cordocentesis is the key standard method of determining hypothyroidism during pregnancy,^4^ but amniocentesis is commonly used to diagnose fetal hypothyroidism due to its complications.^21^ Although hypothyroidism can be diagnosed and treated after birth in a newborn, the disease can possibly affect mental development during the embryo. For this reason, some sources recommend checking fetal thyroid hormones, and treatment is initiated in the case of a diagnosis of fetal goiter during pregnancy. Some sources recommend the intra‐amniotic injection of levothyroxine in a fetus with embryonic goiter.^1^, ^11^, ^22^

Of course, this is not yet approved by international organizations and associations, and the American Thyroid Association (ATA) recommends thyroid hormone measurement even in high‐risk mothers 2‐5 days after birth. One of the recommendations of this association is that in infants who are likely to be born with CH, the neonatal specialist should monitor the condition of the baby before delivery. Further, the American Academy of Pediatrics (AAP) recommends hypothyroidism at a postnatal time, and only in mothers with a history of autoimmune thyroiditis or a previous history of an infant with CH, cordocentesis is considered one of the diagnostic methods but does not emphasize it.^23^ Thus, although studies in Iran show that the prevalence of CH in some parts of the country is ten times more than the global level and has been reported in the range of 1 per 1000 to 1 per 375 neonates,^23^ monitoring fetal hormones and intrauterine treatment have not yet been confirmed for high‐risk mothers.

Fetal endocrine system functions largely independent of the mother but maternal endocrine disorder can influence the fetus adversely. A relationship between GDM and thyroid hormone exist but yet to be established. The finding of raised TSH in GDM fetus as compared to control shows that it could not be due to immaturity of fetal HPT axis or transient hypothyroidism of prematurity which is not possible as gestational age was appropriate (37 weeks). There are studies which demonstrated that pregnant women with increased risk of GDM have a high prevalence of raised ThyAb (thyroid antibody) than observed in general women population. However, few studies have reported no association between diabetes in pregnancy and thyroid function (ThyAb) in pregnant women with GDM.^17^, ^18^

Considering that our study was one of the first CG studies in Iran and the Middle East, further studies are needed to investigate the incidence of this disorder in neonates. As in the present study, the mother had a history of two miscarriages, and in the study of Mirsaeid Ghazi et al, the first case resulted in death and the second case underwent the treatment, and further studies in mothers who have had CG pregnancies or mothers who have had miscarriages for unknown reasons may be added to CG examinations.

## CONFLICT OF INTEREST

None declared.

## AUTHOR CONTRIBUTIONS

SHM: contributed to study concept and design, critically revised the manuscript, and took responsibility for the integrity and the accuracy of the data. AM: contributed to study concept and design, critically revised the manuscript, took responsibility for the integrity and the accuracy of the data, and modified the manuscript. DZ: contributed to study concept and design, critically revised the manuscript, and took responsibility for the integrity and the accuracy of the data. FN: prepared draft of the manuscript, performed literature searching, and reviewed and modified the manuscript. ZGH: performed literature searching and supervised the study. All authors: read and approved the final manuscript.

## ETHICS APPROVAL

Institutional review board approval for case report is not required at our institution. To keeping ethical principles, name of the patient was not pointed in the paper and the rights of the subject were protected. The patient received treatment consistent with the current standard of care.

## CONSENT FOR PUBLICATION

Written informed consent was obtained from the infant's parents for publication of this case report.
